# Exploring the Antioxidant, Neuroprotective, and Anti-Inflammatory Potential of Olive Leaf Extracts from Spain, Portugal, Greece, and Italy

**DOI:** 10.3390/antiox12081538

**Published:** 2023-07-31

**Authors:** Jose M. Romero-Márquez, María D. Navarro-Hortal, Tamara Y. Forbes-Hernández, Alfonso Varela-López, Juan G. Puentes, Raquel Del Pino-García, Cristina Sánchez-González, Iñaki Elio, Maurizio Battino, Roberto García, Sebastián Sánchez, José L. Quiles

**Affiliations:** 1 Department of Physiology, Institute of Nutrition and Food Technology “José Mataix Verdú”, Biomedical Research Centre, University of Granada, 18016 Armilla, Spain; romeromarquez@ugr.es (J.M.R.-M.); mdnavarro@ugr.es (M.D.N.-H.); alvarela@ugr.es (A.V.-L.); crissg@ugr.es (C.S.-G.); 2 University Institute of Research in Olive Grove and Olive Oils, University of Jaén, 23071 Jaén, Spain; jpuentes@ujaen.es (J.G.P.); rgarcia@ujaen.es (R.G.); ssanchez@ujaen.es (S.S.); 3 Research and Development Functional Food Centre (CIDAF), Health Science Technological Park, Avenida del Conocimiento 37, 18016 Granada, Spain; rdpinogarcia@cidaf.es; 4 Sport and Health Research Centre, University of Granada, C/Menéndez Pelayo 32, 18016 Granada, Spain; 5 Research Group on Foods, Nutritional Biochemistry and Health, Universidad Europea del Atlántico, Isabel Torres, 21, 39011 Santander, Spain; inaki.elio@uneatlantico.es (I.E.); m.a.battino@staff.univpm.it (M.B.); 6 Department of Clinical Sciences, Polytechnic University of Marche, 60131 Ancona, Italy; 7 International Joint Research Laboratory of Intelligent Agriculture and Agri-Products Processing, Jiangsu University, Zhenjiang 212013, China

**Keywords:** acetylcholinesterase, cyclooxygenase-2, flavonoids, hydroxyoleuropein, hydroxytyrosol, luteolin, oleuropein, p-hydroxybenzoic acid, phenolics, verbascoside

## Abstract

The leaves of the olive tree (*Olea europaea* L.) are one of the major solid wastes from the olive industry. Globally, the European Union is the largest producer of olive by-products, with Spain, Italy, Greece, and Portugal accounting for almost the entire production. Many questions remain to be solved concerning olive leaves (OL), including those related to possible differences in composition and/or biological activities depending on their geographical origin. In the present work, OL from Spain, Italy, Greece, and Portugal have been characterized according to their phytochemical profile, antioxidant capacity, neuroprotective activity, and anti-inflammatory effects. The Spanish and Italian OL samples presented the highest antioxidant and neuroprotective activities, while the Greek OL showed the lowest. These results were strongly associated with the content of oleoside methyl ester and p-hydroxybenzoic acid for the Spanish and Italian samples, respectively, whereas the content of decarboxymethyl elenolic acid dialdehyde form (hydrated) was negatively associated with the mentioned biological activities of the Greek samples. No country-related effect was observed in the anti-inflammatory activity of OL. Comprehensively, this work could provide a useful tool for manufacturers and R&D departments in making environmentally friendly decisions on how OL can be used to generate nutraceutical products based on the composition and origin of this by-product.

## 1. Introduction

The olive grove industry generates large volumes of solid waste from olive trees (*Olea europaea* L.), including branches, seeds, pulp, and olive leaves (OL). These by-products are important players in the environmental impact of olive oil production due to the high water and energy expenditure for their removal, as well as gas and trash generation. The European Union (EU) is the biggest producer of olive by-products worldwide. Spain, Greece, Italy, and Portugal generate nearly 99% of the EU’s production [1,2]. OL are mainly used for animal consumption, biomass production or incineration [3]. Nevertheless, they are rich in numerous bioactive compounds such as secoiridoid (i.e., oleuropein), phenolic alcohols (e.g., hydroxytyrosol and oleoside), flavones (e.g., luteolin and luteolin-7-o-glucoside), and phenolic acids (i.e., verbascoside) that might be used for other applications such as nutraceutical development [4,5]. In particular, OL has demonstrated important biomedical properties such as antiviral, antimicrobial, anti-inflammatory, antioxidant, and anti-Alzheimer activities [4,6,7]. Therefore, the exploitation of OL to obtain value-added products enriched in phytochemicals might create new economic opportunities for the olive grove industry and reduce the environmental burden generated by these by-products.

Phenolic compounds are the main class of bioactive substances present in olive by-products, contributing to their health-promoting properties [4,6,7]. Recently, the profile and content of phytochemicals in OL extracts have been linked to their antioxidant capacity [3]. Therefore, the composition, concentration, and extraction yield of these compounds may affect OL applications. Numerous factors have been described as OL phytochemical profile modulators, such as olive cultivar, climate, pruning season, drying conditions, and the extraction solvent, among others [4]. However, many questions remain to be solved concerning OL, including those related to possible differences in composition and/or biological activities depending on their geographical origin.

The cholinergic hypothesis of cognitive dysfunction suggests that alterations of acetylcholine-containing neurons in the brain promote the cognitive decline observed during aging and neurodegenerative diseases (ND) [8]. In the same way, the pro-inflammatory process has been to a significantly increased risk of developing ND [9,10,11]. In this context, the intake of olive by-products such as OL has been associated with a reduction in cognitive impairment and inflammation both in vitro and in vivo [4]. Some in vitro tests, such as the inhibitory capacity of acetylcholinesterase (AChE) and cyclooxygenase-2 (COX-2), allow large-scale evaluation of the neuroprotective and anti-inflammatory effects of compounds of interest. Accordingly, the present study evaluated for the first time the direct influence of the geographical origin on the phytochemical profile, antioxidant activity, and neuroprotective and anti-inflammatory effects of OL from Spain, Portugal, Italy, and Greece.

## 2. Materials and Methods

### 2.1. Chemicals and Reagents

All reagents and chemicals were analytical grades. Trizma base, hydrochloric acid, 5,5′-dithiobis-(2-nitrobenzoic acid), physostigmine, acetylthiocholine iodide, and acetylcholinesterase were purchased from Merck (Darmstadt, Germany). The COX-2 Inhibitor Screening Kit was purchased from Abcam (Cambridge, UK). LC-MS grade acetonitrile, LC-MS grade methanol, ethanol, dimethyl sulfoxide, catechin, sodium nitrite, aluminum chloride, sodium hydroxide, gallic acid, 2,2-difenil-1-picrilhidrazil (DPPH), iron (III) chloride hexahydrate, and formic acid used were purchased from Sigma-Aldrich (St. Louis, MO, USA). Folin-Ciocalteu reagent, sodium acetate solution, sodium carbonate, and 2,4,6-tripyridyl-s-triazine (TPTZ) were purchased from Thermo Fisher (Waltham, MA, USA). Finally, 2,2′-azino-di-(3-ethylbenzthiazoline sulfonic acid) was purchased from Roche (Basel, Switzerland), and the water used was Milli-Q type obtained from Millipore purification equipment (Billerica, MA, USA).

### 2.2. Olive Leaves Collection, Classification, and Extraction

For this study, only OL from the so-called “line of flight” were used, that is, the leaves that are collected along with the olives during the harvesting procedure. Leaves from the ground or leaves collected directly from the tree were not included. Therefore, it is a true by-product derived from olive harvesting that inevitably reaches the mill and for which it would be useful to find an application. Forty-nine olive leaf samples from four different countries were chosen for the present study. The 49 OL samples were distributed according to their geographical origin into Spain (n = 26), Italy (n = 6), Greece (n = 8), and Portugal (n = 9) (Table 1).

**Figure 1:**
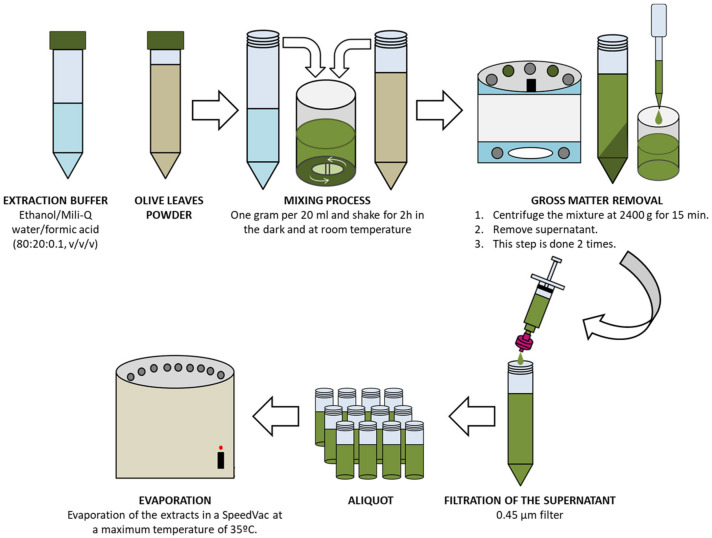
Schematic extraction process of the 49 OL samples. OL = olive leaves.

All samples were harvested between mid-November 2019 and mid-February 2020. Fresh OL were dried at room temperature, ground by a blade mill, and passed through a mesh sieve (<600 μm diameter) to obtain a fine powder. Then, plant material was stored in sealed bags at −20 °C until extraction. The extraction procedure was performed according to the flowchart shown in Figure 1. One gram (1.00 g ± 0.01 g) of dried OL powder was mixed with 20 mL of the extraction buffer (ethanol/Mili-Q water/formic acid, 80:20:0.1, *v*/*v*/*v*) and stirred for two hours in the dark at room temperature. Then, the liquid OL mixture was centrifuged twice at 2400× *g* for 15′, and the supernatants recovered. Supernatants were filtered using a 0.45 μm syringe filter (PBI International, Mejaniga, Italy), aliquoted, evaporated using a Speedvac SC110A (New York, NY, USA), and stored at −80 °C until analyses [12]. The extraction yield was calculated using the Kleeb Bua Daeng formula: yield extraction (%) = M1/M2 × 100, where M1 is the weight of the extract and M2 is the weight of the sample unextracted [13].

### 2.3. Quantification of the Phytochemical Compounds via HPLC-ESI-QTOF-MS/MS Analysis

HPLC analyses were performed on an Agilent 1260 HPLC instrument (Santa Clara, CA, USA) equipped with a binary pump, an online degasser, an auto-sampler, a thermostatically controlled column compartment, as well as a diode array detector. The samples were separated on an Agilent Zorbax Eclipse Plus C18 column (1.8 μm, 4.6 × 150 mm). The mobile phases consisted of water with 0.1% formic acid (A) and methanol with 0.1% formic acid (B) using a gradient elution according to the following profile: 0 min, 5% B; 5 min, 75% B; 10 min, 100% B; 18 min, 100% B; 25 min, 5% B. The initial conditions were maintained for 5 min. The flow rate was 0.8 mL/min, the column temperature was 30 °C, and the injection volume was 5 μL. The compound concentrations of each OL extract were determined by using the area of each individual compound and by interpolation in the corresponding calibration curve. Oleuropein, hydroxytyrosol, luteolin, luteolin-7-O-glucoside, verbascoside, citric acid, and sorbitol were quantified by the calibration curves obtained from their respective commercial standards. The remaining compounds were tentatively quantified based on calibration curves from other compounds with structural similarities. Results are mentioned in tables as mean ± standard deviation (SD).

### 2.4. Total Phenolic Compounds

The total phenolic compounds (TPC) were determined according to the Folin-Ciocalteu colorimetric method with some modifications [14]. Briefly, OL extracts and standards (gallic acid) were mixed with Folin-Ciocalteu reagent for 5 min in a 96-well plate. Then, sodium carbonate was added to each well, stirred, and incubated at room temperature for two hours in the dark. Finally, absorbance was measured at 760 nm in a microplate reader, Synergy Neo2 (Winooski, VT, USA). Results were expressed as milligrams (mg) of gallic acid equivalent (GA) per gram (g) of dry weight (DW) extract. Data are mentioned in the tables as mean ± SD.

### 2.5. Total Flavonoids Content

The total flavonoid content (TFC) was determined according to the aluminum chloride colorimetric method with some modifications [15]. Briefly, OL extracts and standards (catechin) were mixed with sodium nitrite for 6 min in a 96-well plate. Then, aluminum chloride was added and incubated at room temperature for 5 min in the dark. Finally, sodium hydroxide was added to each well, and the absorbance was measured at 510 nm in a microplate reader, Synergy Neo2 (Winooski, VT, USA). Results were expressed as mg of catechin equivalents (CAT)/g of DW extract. Data are mentioned in tables as “mean ± SD”.

### 2.6. Total Antioxidant Activity

The total antioxidant capacity (TAC) of the studied OL was determined using three different methods based on electron transfer (ET). The ET-based methods analyze the ability of a specific antioxidant to reduce an oxidant, changing the color during this reaction. The magnitude of the color change is directly associated with the antioxidant concentration in the sample [16]. In this context, ABTS, FRAP, and DPPH methods were performed following the modified protocols described by Navarro-Hortal et al. (2022), Rivas-García et al. (2022), and Romero-Márquez et al. (2022) [17,18,19]. Results were expressed as μM of Trolox/g of DW extract for the three methods. Data are mentioned in tables as “mean ± SD”.

### 2.7. Acetylcholinesterase (AChE) Inhibition Assay

The AChE inhibitory activity of OL was determined by using the colorimetric method proposed by Ellman et al. (1961), with some modifications [20]. First, OL extracts (1000 μg/mL) were incubated with AChE (10 mU/mL) and 5,5′-dithiobis-(2-nitrobenzoic acid) (150 μM) in 96-plate wells for 15 min at 30 °C. Then, the acetylthiocholine iodide (substrate) was added, and the AChE activity was determined by measuring the changes in the absorbance at 405 nm in a Synergy 2 Biotek plate reader for 25 min at 30 °C. The AChE inhibitory activities were expressed as the mean percentage of inhibitory activity with respect to the positive control’s ± standard error of the mean (SEM).

### 2.8. Cyclooxygenase-2 (COX-2) Inhibition Assay

The COX-2 inhibitory capacity of OL was determined via the Biovision COX-2 Inhibitor Screening Kit (Milpitas, CA, USA), following the manufacturer’s instructions. First, OL extracts (1 μg/mL) were incubated with arachidonic acid/sodium hydroxide solution and the reaction mix (COX-2 human recombinant enzyme, COX cofactor, COX probe, and COX assay buffer) in black 96-plate wells at 37 °C on a Synergy 2 Biotek plate reader for 8 min. The fluorescent signal was registered at 535 nm for extinction and 587 nm for emission. The COX-2 inhibitory activities were expressed as the mean percentage of inhibitory activity with respect to the positive control ± SEM.

### 2.9. Statistical Analysis

The experimental procedures were performed at least three times. The statistical software IBM SPSS 25 (Chicago, IL, USA) was used for the analysis of normality, variance homogeneity, analysis of variance (ANOVA), and Pearson’s correlation analysis. ANOVA was carried out, and the *post hoc* HSD Tukey multiple range test was considered significant when *p* < 0.05. Principal component analysis (PCA) and the correlation heatmap were performed using MetaboAnalyst V5.0, using the information obtained from the mean values of the 49 samples, including TPC, TFC, TAC (ABTS, FRAP, and DPPH), the inhibitory activity of AChE and COX-2, as well as the 52 compounds identified after HPLC-ESI-QTOF-MS/MS analysis.

## 3. Results and Discussion

### 3.1. TPC, TFC and TAC

The total content of phenolic compounds and flavonoids, as well as the extraction yield of OL with respect to the different countries, can be observed in Table 2. The Italian OL had the lowest extraction yield. However, they presented the highest values of TPC and TFC (40.98 ± 5.01 mg GA/g DW and 28.56 ± 6.75 mg CAT/g DW, respectively), followed by the Portuguese samples (37.1 ± 4.2 mg GA/g DW and 15.6 ± 9.3 mg CAT/g DW, respectively) and the Greek OL (31.69 ± 2.05 mg GA/g DW and 8.99 ± 2.47 mg CAT/g DW, respectively). When compared with the existing evidence, the TPC of the Spanish and Italian samples was similar, or even higher, than those found in the literature [6,21], whereas the TFC was lower [6,21,22]. Similarly, the TPC and TFC for Greek samples were in accordance with those obtained by Petridis et al. (2012) and Papoti et al. (2018) in OL samples from Greek varieties [23,24]. However, there are no specific data available to compare the TPC and TFC obtained in the Portuguese OL samples since the single study expressed the values in fresh weight [25]. The phenolic and flavonoid content in OL extracts can be modified by numerous factors, such as the harvesting season, drying conditions, and extraction solvent, which can affect the determinations in order to compare between countries [4]. According to literature, harvesting season has been shown to modulate the TPC and TFC, with higher values for leaves cultivated between March and April [22,26]. Similarly, the drying method and the solvent used for OL extraction have been related to changes in the phenolic and flavonoid content [21,27]. Therefore, these procedures were standardized for the 49 samples, with all samples collected between November and February. Likewise, for all samples, room temperature and an ethanol/water mixture were selected as drying and extraction methods, respectively, to reduce the influence of these factors on the determinations.

**Figure 2:**
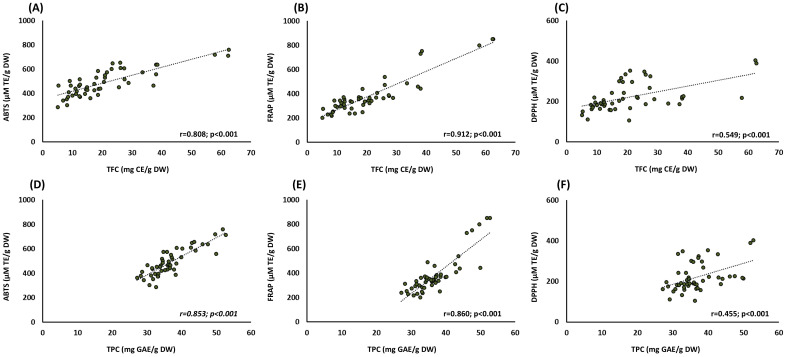
Pearson’s correlation scatterplot of the relationships between (**A**) ABTS and TPC, (**B**) FRAP and TPC, (**C**) DPPH and TPC, (**D**) ABTS and TFC, (**E**) FRAP and TFC, and (**F**) DPPH and TFC. Abbreviations: ABTS: 2.20-azinobis (3-ethylbenzothiazoline-6-sulfonic acid); CE: catechin equivalents; DPPH: 2.2-diphenyl-1-picryl-hydrazyl-hydrate; FRAP: ferric reducing antioxidant power; DW: dry weight; GAE: gallic acid equivalents. Dashed line represents the regression curve.

There are several in vitro assays to determine the TAC of agri-food matrices and their by-products. Most of them are one-electron transfer-based methods, but their sensitivity depends on several factors such as pH, hydrophobic or lipophilic affinity, etc. [16]. Therefore, it is strongly recommended to use at least two different methods for the determination of TAC, particularly when studying such phytochemically complex matrices as OL extracts. In the present work, TAC was assessed by three different methods. For the ABTS assay, the highest value was obtained by the Italian and Spanish OL samples, followed by the Portuguese OL. In contrast, the lowest ABTS score was obtained by the Greek samples. A similar profile was obtained for the FRAP test, with the highest antioxidant capacity for the Italian samples, whereas the Greek samples had the lowest. No statistical differences were obtained for the DPPH assay among samples from different origins. These results can be partially explained due to the content of phenolic compounds and, specifically, flavonoids present in OL, which were directly associated with their antioxidant capacity (Figure 2). According to the obtained data, a strong association was found between TPC and TFC and ABTS and FRAP, whereas the association with DPPH was lower but still statistically significant. These results were also supported by Zhang et al. (2022), who showed a strong association between TFC and ABTS (r = 0.6236; *p* < 0.001) and FRAP (r = 0.6856; *p* < 0.001), whereas DPPH was not significant in 32 OL samples from China [3].

These differences might also be explained by the different methods used to determine antioxidant capacity. To mention, the application of the DPPH method is limited due to the nitrogenous nature of the free radical, as the kinetic reaction is not linear between the free radical and the antioxidant compounds [16]. In addition, some antioxidants can react slowly or even be inert to the DPPH radical, and some reactions with phenolic compounds can be reversible in the DPPH assay [16,28,29]. Therefore, lower antioxidant capacity values can be obtained by this method, which might explain the absence of statistical differences in the literature [3,28]. In contrast, the ABTS and FRAP methods have been shown to be more valid than the DPPH assay for the evaluation of antioxidant activity [16,28].

### 3.2. Phytochemical Compounds Quantification

Table 3 shows the phytochemical profile of OL according to its geographical origin, where 52 compounds were quantified and divided into ten groups.

**Figure 3:**
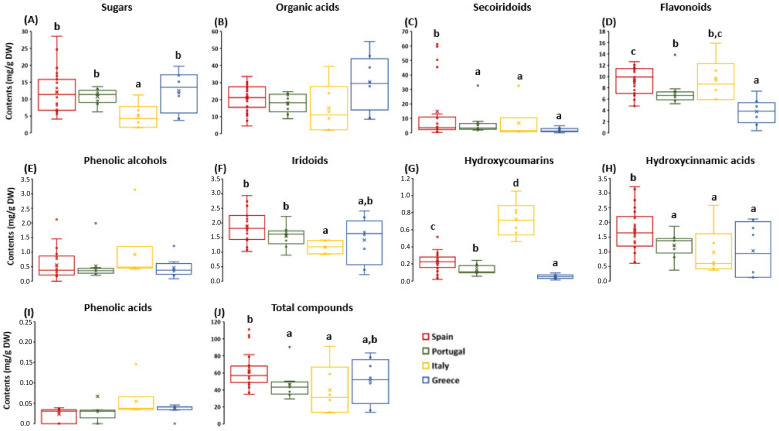
Box and whisker Plot of (**A**) sugars, (**B**) organic acids, (**C**) secoiridoids, (**D**) flavonoids, (**E**) phenolic alcohols, (**F**) iridoids, (**G**) hydroxycoumarins, (**H**) hydroxycinnamic acids, (**I**) phenolic acids, and (**J**) total compounds present in OL from Spain, Italy, Portugal, and Greece. Results are expressed as mean ± SD. For each parameter, different letters indicate statistically significant differences between countries (*p* < 0.05). OL: olive leaves.

Sorbitol was the most abundant sugar in the OL extracts (Table 3), with the Italian samples having the lowest content. Regarding the organic acids, quinic acid was the most abundant, with the Greek samples being the richest in this compound. No statistical differences were found in most of the quantified secoiridoid molecules, but when considering the complete sum of this family of compounds, the Spanish OL stood out above the rest (Figure 3C).

Similar results were observed for the flavonoid content (Figure 3D). In this case, the Spanish and Italian samples presented the highest flavonoid content, highlighting the content in luteolin and its derivatives (luteolin glucoside and luteolin-7-O-glucoside), as well as apigenin and its derivatives (apigenin-7-O-glucoside and apigenin-7-O-rutinoside). The Italian samples, but not the Spanish ones, showed the highest content of hydroxytyrosol (Table 3). In the same way, other phenolic compounds, such as iridoids (e.g., 7-epiloganin) and hydroxycoumarins (e.g., esculetin), were spotlighted in the Spanish and Italian samples, while lamiol and lauroside B were abundant in the Greek and Portuguese OL. Taken together, the most representative phytochemical compounds in the OL studied in this work, including phenolic alcohols (hydroxytyrosol), flavonoids (luteolin-7-O-glucoside, luteolin-4-O-glucoside, and apigenin-7-O-glucoside), as well as secoiridoids and their derivatives (oleoside and ligstroside), were lower compared to the OL extracts previously reviewed by Romero-Márquez et al. (2023) from Spain, China, Italy, and Turkey. An exception was observed in the oleuropein content, reaching levels compatible with the literature in the Spanish, Italian, and Portuguese OL samples [4].

Although it has been suggested that the olive variety may contribute to the differences in terms of phytochemical composition [26,30], some authors have shown that despite the differences between cultivars, Spanish OL have a higher content of flavonoids and secoiridoids than Italians, indicating a similar effect of geographical origin beyond the variety of OL used [22]. In fact, the pruning season has been demonstrated to be the major influence on the phytochemical profile, even in the same cultivar [22,26]. In the same way, regular annual precipitation and warm summer temperatures, as well as limited pluvial floods, have been associated with an improvement in the phytochemical content of olive trees [31,32]. Therefore, in the context of climate change, there is a strong northwest-southeast gradient in these features in Europe. In particular, Eastern Mediterranean countries such as Greece have reported a reduction in annual precipitation, an increase in summer hot and dry weather, and winter flood conditions [33]. In contrast, south-west Mediterranean countries such as Spain or Italy seem to conserve similar bioclimatic conditions [34]. These results might explain the differences reported in the phytochemical profile regarding Spain, Italy, and Greece. Therefore, in the present work, it has been demonstrated that the geographical origin dramatically influences the content of some compounds such as sugars, secoiridoids, flavonoids, iridoids, hydroxycoumarins, and hydroxycinnamic acids in olive leaves.

### 3.3. Multivariate Data Analysis

#### 3.3.1. Principal Component Analysis

PCA has been extensively used as an unsupervised exploratory method to decrease the dimensionality of datasets. It has been widely used in several research fields, such as microbiome studies, population genetics, epidemiology, and agricultural science [3]. In the present work, it was applied considering the large volume of data/variables analyzed, with the intention of better interpreting the obtained results and visualizing possible relationships among them. In that sense, the PCA was performed by the MetaboAnalyst V5.0 using the information obtained from the 49 samples, including: TPC, TFC, TAC (ABTS, FRAP, and DPPH), the inhibitory activity of AChE and COX-2, as well as the 52 compounds quantified by HPLC-ESI-QTOF-MS/MS.

**Figure 4:**
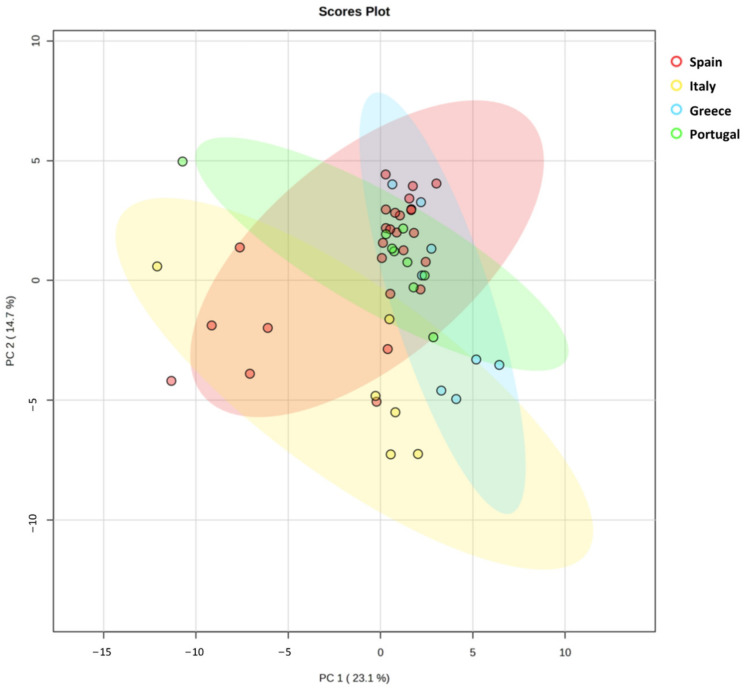
PCA score plot results obtained from the mean values of the seven colorimetric determinations (TPC, TFC, ABTS, FRAP, DPPH, AChE, and COX-2) and the 52 phytochemical compounds present in the OL from Spain, Italy, Greece, and Portugal. OL: olive leaves.

The first two principal components (PCs) described 37.8% of total variance (PC1: 23.1% and PC2: 14.7%) and were used to visualize the relationship between countries. The distribution of the four evaluated countries is shown in Figure 4. As shown in the score plots, PC1 was able to separate the Italian samples from the Greek ones, whereas PC2 was able to separate the Italian samples from the Portuguese OL. Nevertheless, the Spanish OL could not be differentiated as a group due to their high variability and were randomly distributed in Figure 4. Most of the samples from Spain, Greece, and Portugal are distributed in the right part of PC1 and the upper part of PC2, whereas the Italian samples are in the opposite quadrant. An interesting feature was observed concerning the distribution of 7 samples from Spain in a similar way to the Italian OL. The Spanish samples situated in the bottom-left quadrant have in common that they are from Andalusia, the south of Spain. This might be explained due to the similarities between the Italian and Andalusian climates, which exert similar threshold temperatures that have been associated with bioclimatic requirements for olive development [34].

#### 3.3.2. Inhibitory Activity of AChE and COX-2 as Well as Correlation Analysis

**Figure 5:**
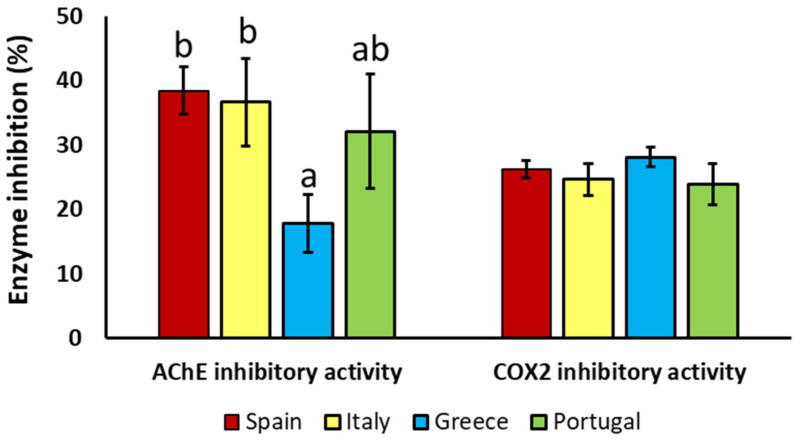
Acetylcholinesterase (AchE) and cyclooxygenase-2 (COX2) inhibitory activity exerted by OL extracts from Spain, Italy, Greece, and Portugal. Results are expressed as the mean ± standard error of the mean. For each of the parameters, bars with different letters indicate statistically significant differences between countries (*p* < 0.05). OL: olive leaves.

The cholinergic hypothesis of cognitive dysfunction suggests that an alteration of acetylcholine concentration in neurons of the brain promotes the cognitive decline observed during aging and numerous NDs, such as Alzheimer’s disease (AD) [8]. In this case, the hyperactivity of some enzymes, such as acetylcholinesterase (AChE), is associated with cognitive dysfunction, favoring the degradation of acetylcholine and promoting the deficit of these neurotransmitters in the brain. These events result in memory loss and other cognitive symptoms related to dementia. Therefore, cholinergic-based strategies have been proposed as a realistic approach to drug development for the treatment of ND. As far as it is known, this is the first study evaluating the inhibitory AChE activity of OL based on geographical origin on a large scale. As can be observed in Figure 5, the highest inhibitory AChE activity was exhibited by the Spanish (38.46 ± 3.72) and the Italian (36.64 ± 6.76) samples. In contrast, the lowest AChE inhibitory activity was observed by the Greek OL (17.81 ± 4.53). These differences might be attributed to differences in the aforementioned phytochemical profile.

**Figure 6:**
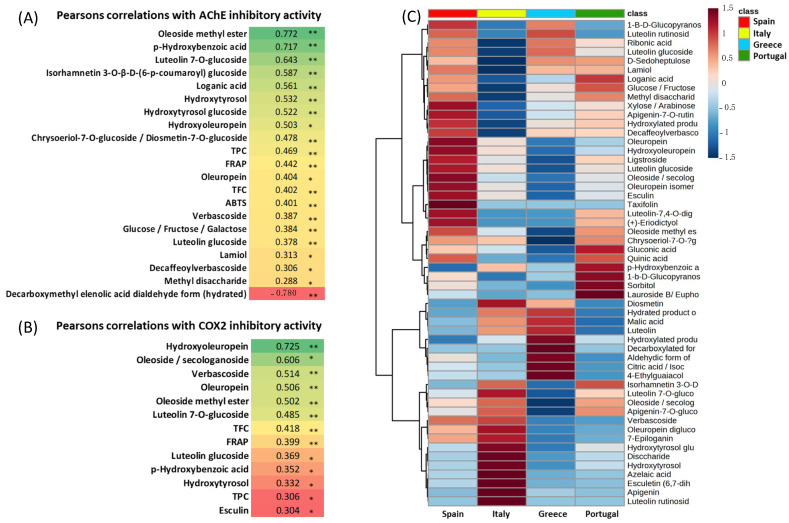
Pearson’s correlation analysis of the relationships between (**A**) AChE inhibitory activity and phytochemical compounds, (**B**) COX-2 inhibitory activity and phytochemical compounds. (**C**) Heatmap analysis exposing the relative amount (red is higher and blue is lower) of the specific phytochemical compound based on geographical origin. * means *p* < 0.05 and ** means *p* < 0.01. Abbreviations: ABTS: 2.20-azinobis (3-ethylbenzothiazoline-6-sulfonic acid); FRAP: ferric reducing antioxidant power; TFC: total flavonoids content; TPC: total phenolic compounds.

Pearson’s correlation analysis showed that AChE inhibitory activity may be influenced by numerous compounds in the OL from different origins. As shown in Figure 6A, the AChE inhibitory activity of the Spanish samples was strongly associated with the oleoside methyl ester content. In the same way, loganic acid, hydroxyoleuropein, chrysoeriol-7-O-glucoside, and oleuropein content were moderately associated with the neuroprotective activity exerted by the Spanish samples. Verbascoside, glucose, luteolin glucoside, lamiol, decaffeoylverbascoside, and methyl disaccharide were slightly associated with the anti-AChE in the Spanish OL samples (Figure 6A,C). Similarly, the anti-AChE activity exerted by the Italian samples was associated differently with their specific phytochemical profile. A strong association was found between p-hydroxybenzoic acid and anti-AChE activity. In the same way, other compounds such as luteolin-7-O-glucoside, isorhammentin-3-O-β-D-(6-p-coumaroyl) glucoside, and hydroxytyrosol and its derivatives were moderately correlated with the neuroprotective activity exerted by the Italian samples. The anti-cholinergic activity of the Portuguese samples was associated with oleoside methyl ester in a similar way to the Spanish samples, indicating a possible influence of the geographical region on the anti-cholinergic activity promoted by this compound (Figure 6A,C). Nevertheless, despite the different correlations with specific compounds, no statistical differences were found between the anti-AChE activity of the Spanish, Italian, and Portuguese samples. This effect might be attributed to the fact that the total phenolic content is similar among samples. Therefore, the specific individual phytochemical compound contribution to the anti-cholinergic activity might be masked due to synergistic activity with the rest of the compounds, as previously described [4]. However, the Greek samples exhibited the lowest anti-cholinergic activity compared to the rest. In this case, an interesting result concerning the neuroprotective effect was found. A strong negative correlation was observed between the high content of the hydrated decarboxymethyl elenolic acid dialdehyde form in the Greek samples and the anti-cholinergic activity exerted (Figure 6A). This association and the low phenolic content might explain, at least in part, the low AChE inhibitory activity exerted by the Greek samples. Up to date, this is the first research that has evaluated the country-related neuroprotective effect of OL on a large scale and opened the door for further research for the development of cholinergic-based nutraceutical formulations using local olive leaves. It should be highlighted that despite the similar neuroprotective effect exerted by the Spanish, Italian, and Portuguese OL, the low extraction yield obtained by the Italian samples might limit their use as potential nutraceutical formulations, requiring higher amounts of OL to achieve profitable extract production.

On the other hand, the anti-inflammatory effect of OL was explored by analyzing the in vitro inhibitory activity of COX-2. COX-2 is a highly inducible enzyme required to produce prostaglandins, which are involved in the inflammatory process [35]. Selective nonsteroidal anti-inflammatory drugs inhibit COX-2, which has been associated with a marked reduction in the risk of developing NDs such as AD [9,10,11]. However, the increased intake of these selective COX-2 inhibitors has been associated with an increase in side effects during aging [36]. Therefore, the use of natural compounds for the formulation of COX-2 inhibitor-based strategies might contribute to drug development for the treatment of inflammation related to ND. Despite the remarkable anti-inflammatory activity exerted by the different OLs, no origin-related effect was observed (Figure 5). Pearson’s correlations indicate that the major contribution to the anti-inflammatory activity exerted by OL was attributed to hydroxyoleuropein, oleoside, verbascoside, oleuropein, oleoside methyl ester, and luteolin-7-O-glucoside content (Figure 6B). These results are in accordance with the literature, which showed that OL [37,38], and numerous isolated compounds such as oleuropein and its derivatives [38,39], verbascoside [40], and luteolin-7-O-glucoside [41] were able to reduce COX-2 activity. However, there is not enough information in the current literature about the anti-inflammatory effect of oleosides and their derivatives, and this work provides the opportunity for further research to evaluate the potential biomedical properties of these compounds.

## 4. Conclusions

The present work evaluated the influence of the geographical origin on the phytochemical profile, antioxidant activity, and neuroprotective and anti-inflammatory effects of OL from Spain, Portugal, Greece, and Italy. The results pointed out that the geographical origin considerably influenced the phytochemical profile, especially in relation to the content of sugars, secoiridoids, flavonoids, iridoids, hydroxycoumarins, and hydroxycinnamic acids. The antioxidant capacity was associated with the total content of phenolic compounds and flavonoids, which stood out in the samples from Spain and Italy. It was also shown that the inhibitory acetylcholinesterase activity was influenced by several compounds present in the OL, with the Spanish, Italian, and Portuguese samples being the most interesting for this purpose. Despite the remarkable ability to inhibit cyclooxygenase-2 activity exerted by the different OLs, no origin-related effect was observed for this marker among the investigated countries. It should be highlighted that the extraction yield of the Italian samples was lower, which might condition their use as potential nutraceutical formulations by requiring higher amounts of OL to achieve profitable extract production. These results might indicate that not every OL can be used for different purposes and that it might be used according to its phytochemical profile. As limitations of the study, it can be mentioned that the in vitro tests for evaluating the AChE and COX-2 inhibitory activity allow the analysis of the large-scale effect of numerous food by-products but do not take into account the complexity of the biological systems and the potential interaction of the enzyme studied with other cellular components, which could influence the inhibitory activity of the compounds studied in vivo. Likewise, the multivariate data analysis applied is not able to find a causal relationship between the variables studied. However, it is a robust tool for generating a concept map of the observed effect.

Comprehensively, this work provides useful information for manufacturers and R&D departments to make environmentally friendly decisions on how OL can be used to generate nutraceutical products based on the composition and origin of this by-product.

## Figures and Tables

**Table 1:** Ethical Statements of the OL from four different countries.

Provider Institution	Region	Country	n
CRDOP Estepa	Seville	Spain	13
ACE	Jaen	Spain	7
IRTA	Barcelona	Spain	6
Pugliaolive	Bari	Italy	4
Parma University	Parma	Italy	2
NGC	Peloponnese	Greece	6
ACK	Kalamata	Greece	2
CEPAAL	Alentejo	Portugal	7
Esporão	Alentejo	Portugal	2

Abbreviations: ACE: Almazara Cruz de Esteban; ACK: Agricultural Cooperative of Kalamata; CEPAAL: Centro de Estudos e Promoção do Azeite do Alentejo; CRDOP Estepa: Consejo Regulador Denominación de Origen Protegida Estepa; IRTA: Instituto de investigación y tecnología agroalimentarias; NGC: NILEAS Producers Group Company A.C. OL: olive leaves.

**Table 2:** TPC, TFC and TAC of OL extracts from Spain, Italy, Greece, and Portugal.

Determination	Spain	Italy	Greece	Portugal
Yield (%)	26.2 ± 5.4 ^ab^	19.5 ± 10.4 ^a^	29.1 ± 4.8 ^b^	29.2 ± 5.7 ^b^
TPC (mg GA/g DW)	37.8 ± 6.8 ^ab^	41.0 ± 5.0 ^b^	31.7 ± 2.0 ^a^	37.1± 4.2 ^ab^
TFC (mg CAT/g DW)	24.7 ± 15.2 ^b^	28.6 ± 6.7 ^b^	9.0 ± 2.5 ^a^	15.6 ± 9.3 ^ab^
ABTS (μM Trolox/g DW)	517 ± 108 ^bc^	591 ± 60 ^c^	372 ± 61 ^a^	459 ± 78 ^ab^
FRAP (μM Trolox/g DW)	398 ± 169 ^ab^	505± 124 ^b^	266 ± 60 ^a^	362 ± 151 ^ab^
DPPH (μM Trolox/g DW)	240 ± 82	226 ± 55	168 ± 32	217 ± 51

Abbreviations: ABTS: 2.20-azinobis (3-ethylbenzothiazoline-6-sulfonic acid); CE: catechin equivalents; DPPH: 2.2-diphenyl-1-picryl-hydrazyl-hydrate; FRAP: ferric reducing antioxidant power; DW: dry weight; GAE: gallic acid equivalents. OL: olive leaves. TFC: total flavonoids content. TPC: total phenolic compounds. TAC: total antioxidant capacity. Results are expressed as mean ± SD. For each determination, different letters indicate statistically significant differences between countries (*p* < 0.05).

**Table 3:** Phytochemical identification and quantification of the OL extracts from Spain, Italy, Greece, and Portugal.

Compound	Formula	*m*/*z*	Error (ppm)	Spain	Italy	Greece	Portugal
**Sugars**							
Sorbitol	C6H14O6	181.0723	−2.61	9.76 ± 5.82 ^b^	3.47 ± 3.4 ^a^	10.16 ± 5.57 ^b^	8.39 ± 2.35 ^ab^
D-Sedoheptulose	C7H14O7	209.0671	−1.91	0.53 ± 0.2	0.35 ± 0.21	0.69 ± 0.31	0.58 ± 0.11
D-glucose/D-fructose/D-galactose	C6H12O6	179.0563	−0.73	0.76 ± 0.21 ^b^	0.36 ± 0.14 ^a^	0.74 ± 0.27 ^b^	0.77 ± 0.22 ^b^
D-xylose/L-arabinose	C5H10O5	149.0456	−0.35	0.31 ± 0.09	0.19 ± 0.07	0.25 ± 0.14	0.27 ± 0.05
Disccharide	C12H20O10	323.0982	0.6	0.28 ± 0.07 ^ab^	0.33 ± 0.1 ^b^	0.23 ± 0.11 ^a^	0.27 ± 0.06 ^ab^
Methyl disaccharide	C11H20O9	295.1036	−0.3	0.5 ± 0.15 ^a^	0.27 ± 0.21 ^b^	0.39 ± 0.18 ^ab^	0.41 ± 0.07 ^ab^
**Organic acids**							
Gluconic acid	C6H12O7	195.0517	−3.43	8.02 ± 4.38	5.4 ± 6.53	10.23 ± 8.1	5.14 ± 3.29
Ribonic acid	C5H10O6	165.041	−3.22	3.89 ± 1.34 ^b^	1.46 ± 0.98 ^a^	3.55 ± 1.76 ^b^	3.6 ± 0.77 ^b^
Quinic acid	C7H12O6	191.0567	−2.85	8.4 ± 4.92 ^a^	6.61 ± 6.39 ^a^	13.87 ± 7.06 ^b^	8.84 ± 3.87 ^a^
Malic acid	C4H6O5	133.0148	−4.03	0.97 ± 0.75	3.51 ± 1.42	2.93 ± 1.33	0.97 ± 0.33
Citric acid/Isocitric acid	C6H8O7	191.0201	−1.63	1.03 ± 0.75	0.78 ± 0.19	1.7 ± 1.07	0.39 ± 0.19
**Secoiridoids**							
Oleuropein	C25H32O13	539.177	0.2	10.27 ± 18.69	4.72 ± 9.04	0.11 ± 0.06	2.69 ± 6.61
1-β-D-Glucopyranosyl acyclodihydroelenolic acid isomer 1	C17H28O11	407.1562	−0.42	0.37 ± 0.17	0.32 ± 0.13	0.4 ± 0.22	0.33 ± 0.19
Decarboxymethyl elenolic acid dialdehyde form isomer 1 (Hydroxylated)	C10H14O5	213.0769	0.09	0.19 ± 0.1 ^a^	-	0.18 ± 0.03 ^b^	0.16 ± 0.09 ^a^
Oleoside/secologanoside	C16H22O11	389.109	−0.05	1.61 ± 1.13	-	-	0.93 ± 1.03
1-β-D-Glucopyranosyl acyclodihydroelenolic acid isomer 2	C17H28O11	407.1561	−0.23	0.67 ± 0.37 ^a^	0.18 ± 0.07 ^a^	0.67 ± 0.45 ^ab^	1.21 ± 0.76 ^b^
Decarboxymethyl elenolic acid dialdehyde form (Hydrated)	C9H14O5	201.0772	−1.63	0.15 ± 0.17	0.11 ± 0.07	0.32 ± 0.14	-
Decarboxymethyl elenolic acid dialdehyde form isomer 2 (Hydroxylated)	C9H12O5	199.0615	−1.21	0.28 ± 0.14	0.33 ± 0.17	0.67 ± 0.8	0.42 ± 0.33
Decarboxylated form of hydroxy elenolic acid isomer 2	C10H14O5	213.0768	0.11	-	-	0.5 ± 0.12	-
Oleoside methyl ester	C17H24O11	403.1243	0.94	1.03 ± 1.18	0.46 ± 0.6	-	0.7 ± 0.72
Aldehydic form of decarboxymethyl elenolic acid	C10H16O5	215.0929	−1.69	0.24 ± 0.19	0.13 ± 0.12	0.51 ± 0.52	0.07 ± 0.01
Hydroxyoleuropein	C25H32O14	555.1712	1.45	0.48 ± 0.58	0.52 ± 0.26	-	0.54 ± 0.56
Oleuropein diglucoside	C31H42O18	701.2288	1.85	0.31 ± 0.12	-	-	-
Oleuropein isomer	C25H32O13	539.1767	0.78	3.15 ± 2.27	1.19 ± 1.67	-	-
Ligstroside	C25H32O12	523.1815	1.36	0.86 ± 0.12	-	-	-
**Flavonoids**							
Luteolin-7,4-O-diglucoside/Rutin	C27H30O16	609.1451	1.84	0.15 ± 0.05	-	-	0.07 ± 0.05
Luteolin rutinoside isomer 2	C27H30O15	593.1505	1.39	0.1 ± 0.05	-	0.07 ± 0.05	-
Luteolin-7-O-glucoside	C21H20O11	447.0934	0.12	1.14 ± 0.67	1.74 ± 2.37	0.62 ± 0.53	1.1 ± 1.22
Apigenin-7-O-rutinoside	C27H30O14	577.1559	0.7	0.52 ± 0.22 ^b^	0.18 ± 0.05 ^a^	0.32 ± 0.11 ^a^	0.34 ± 0.08 ^ab^
Taxifolin	C15H12O7	303.0509	0.52	0.04 ± 0.04	-	-	-
Apigenin-7-O-glucoside	C21H20O10	431.0981	0.87	0.29 ± 0.16	0.5 ± 0.36	0.09 ± 0.04	0.38 ± 0.13
Luteolin glucoside	C21H20O11	447.0934	0.06	4.96 ± 2.06 ^b^	2.9 ± 2.06 ^ab^	1.28 ± 0.93 ^a^	3.43 ± 1.19 ^ab^
Chrysoeriol-7-O-glucoside/Diosmetin-7-O-glucoside	C22H22O11	461.1086	0.86	0.62 ± 0.16 ^b^	0.53 ± 0.35 ^ab^	0.27 ± 0.15 ^a^	0.67 ± 0.16 ^ab^
Azelaic acid	C9H16O4	187.0979	−1.55	0.5 ± 0.36 ^a^	2.02 ± 1.47 ^b^	0.13 ± 0.05 ^a^	0.27 ± 0.1 ^a^
Luteolin glucoside	C21H20O11	447.0932	0.46	0.45 ± 0.12 ^b^	0.23 ± 0.07 ^a^	0.45 ± 0.24 ^ab^	0.4 ± 0.11 ^ab^
(+)-Eriodictyol	C15H12O6	287.0565	−1.05	0.19 ± 0.09 ^b^	-	-	0.08 ± 0.08 ^a^
Isorhamnetin-3-O-β-D-(6-p-coumaroyl) glucoside	C31H28O14	623.1395	1.94	0.03 ± 0.02	0.1 ± 0.12	0.04 ± 0.05	0.08 ± 0.08
Luteolin	C15H10O6	285.0412	−2.43	0.27 ± 0.19	0.38 ± 0.18	0.43 ± 0.3	0.19 ± 0.08
Apigenin	C15H10O5	269.046	−1.49	0.14 ± 0.08 ^a^	0.73 ± 0.27 ^b^	0.18 ± 0.16 ^a^	0.12 ± 0.08 ^a^
Diosmetin	C16H12O6	299.0562	−0.32	0.19 ± 0.09 ^a^	0.51 ± 0.13 ^c^	0.32 ± 0.16 ^b^	0.16 ± 0.07 ^a^
Luteolin rutinoside isomer 1	C27H30O15	593.1502	1.79	-	0.03 ± 0.02	-	-
**Phenolic alcohols**							
Hydroxytyrosol	C8H10O3	153.0556	−0.84	0.12 ± 0.09 ^a^	0.35 ± 0.13 ^b^	0.05 ± 0.01 ^a^	0.12 ± 0.13 ^a^
Hydroxytyrosol glucoside	C14H20O8	315.1086	−0.08	0.36 ± 0.46	0.50 ± 1	0.36 ± 0.38	0.33 ± 0.45
4-Ethylguaiacol	C9H12O2	151.0764	0.36	0.1 ± 0.06	0.09 ± 0.02	0.15 ± 0.11	0.08 ± 0.03
**Iridoids**							
Loganic acid	C16H24O10	375.1297	0.15	0.26 ± 0.06 ^b^	0.11 ± 0.11 ^a^	0.13 ± 0.09 ^a^	0.26 ± 0.1 ^b^
7-Epiloganin	C17H26O10	389.1457	−0.58	0.7 ± 0.21 ^ab^	0.98 ± 0.35 ^b^	0.54 ± 0.43 ^a^	0.51 ± 0.22 ^a^
Lamiol	C16H26O10	377.146	−1.34	0.93 ± 0.34 ^b^	0.1 ± 0.06 ^a^	0.74 ± 0.66 ^b^	0.79 ± 0.29 ^b^
**Hydroxycoumarins**							
Esculetin	C9H6O4	177.0198	−2.32	0.14 ± 0.06 ^b^	0.68 ± 0.19 ^c^	0.05 ± 0.02 ^a^	0.1 ± 0.04 ^ab^
Esculin	C15H16O9	339.0721	0.23	0.08 ± 0.05	0.04 ± 0.03	-	0.04 ± 0.02
**Hydroxycinnamic acid**							
Verbascoside	C29H36O15	623.1974	1.41	0.41 ± 0.52	0.38 ± 0.25	0.08 ± 0.04	0.18 ± 0.14
Decaffeoylverbascoside	C20H30O12	461.1665	0.19	1.33 ± 0.86	0.6 ± 0.64	0.96 ± 0.93	1.04 ± 0.36
**Phenolic acids**							
p-Hydroxybenzoic acid	C7H6O3	137.0247	−1.67	-	0.05 ± 0.04	-	0.09 ± 0.14
**Other compounds**							
Lauroside B/Euphorbioside A	C19H32O9	403.1972	0.48	0.28 ± 0.16 ^a^	0.3 ± 0.29 ^ab^	0.37 ± 0.29 ^ab^	0.71 ± 0.39 ^b^

Results are expressed as milligrams of compound (mean ± SD) per gram of dry weight. For each compound, different letters between countries indicate statistically significant differences (*p* < 0.05). The symbol (-) was added when the specific compound was not detected. *m*/*z*: mass-to-charge ratio. OL: olive leaves. ppm: parts per million.

## Data Availability

The data presented in this study are available on request from the corresponding author. The data are not publicly available yet because funded grants are still ongoing.
